# Editorial: The Role of Breast Cancer Stem Cells in Clinical Outcomes

**DOI:** 10.3389/fonc.2020.00299

**Published:** 2020-03-10

**Authors:** Dayanidhi Raman, Amit K. Tiwari, Venkataswarup Tiriveedhi, Julie A. Rhoades (Sterling)

**Affiliations:** ^1^Department of Cancer Biology, University of Toledo Health Science Campus, Toledo, OH, United States; ^2^Department of Pharmacology and Experimental Therapeutics, University of Toledo Health Science Campus, Toledo, OH, United States; ^3^Department of Biological Sciences, Tennessee State University, Nashville, TN, United States; ^4^Department of Medicine, Vanderbilt University Medical Center, Nashville, TN, United States; ^5^Biomedical Engineering, Vanderbilt University Medical Center, Nashville, TN, United States

**Keywords:** breast cancer stemness, metabolic plasticity, tumor heterogeneity, tumor microenvironment, BCSC niche, chemoresistance, immunotherapy

Breast cancer stem cells (BCSCs) or tumor-initiating cells (TICs) constitute a small, dynamic subpopulation of breast tumors and are important drivers of tumor initiation, progression and metastasis [Sridharan, Howard et al., (1, 2)]. They play a vital role in constitutive or acquired drug resistance or chemoresistance that results in poor patient outcomes (3). Following conventional cytotoxic or radiotherapy, the inherently resistant and surviving BCSCs, non-BCSC tumor and stromal cells constitute the minimal residual disease (MRD) (3, 4). Subsequently, these BCSCs expand and undergo multilineage differentiation and repopulate the heterogeneous tumor. The relapsed tumor is highly aggressive, probably cross-drug resistant and highly metastatic, posing grave prognosis. BCSCs possess innate or develop acquired chemoresistance as they have the ability to detoxify or transport out the chemotherapeutic drug through a variety of mechanisms. This directs our focus to either selectively target BCSCs or co-target BCSCs and non-BCSCs (bulk tumor cells) to overcome chemoresistance and achieve clinical success in metastatic breast cancer (MBC) patients. Vulnerable targets or signaling nodes should be identified and targeted in BCSCs. Importantly, the biological characteristics, molecular pathways that sustain stemness, induction of resistance mechanisms, facilitation of the plasticity of the BCSCs should be ascertained (5). Mathematical modeling approaches may be employed to discern the behavior of the BCSCs and its niche (6, 7). Additionally, nanotechnology and targeted delivery of drugs must be developed and tailored to improve the drug efficacy and minimize the adverse events in the patients.

In this special guest edition, Zhou et al., have elegantly reviewed cellular origins of the breast cancer to understand heterogeneity, a variety of BCSC markers, regulatory signaling pathways, microRNAs and therapeutic strategies in different subtypes of BC. BCSCs also rewire their energetics to bolster their survival (8). Energetic CSCs are a subset of BCSCs that demonstrate increased proliferative capacity, enhanced anchorage-independent growth and aldehyde dehydrogenase positivity (9, 10). Walsh et al., have reviewed the various factors that enable the BCSCs to flick the energetic switch to gain metabolic plasticity. Targeting such metabolic vulnerabilities would be a powerful approach to combat BC stemness. Redox processes also play a vital role in the viability of BCSCs to detoxify xenobiotics and control the level of reactive oxygen species. Recent evidence suggests that by targeting the redox status, the mesenchymal state of BCSCs can be switched to epithelial type which is comparatively more susceptible to cytotoxic drugs (11). BCSCs also evade the immune system but they are strongly antigenic as per Khandekar et al., enabling naïve CD8^+^ effector T-cells to eliminate BCSCs. The challenge would be in a situation where CD8^+^ T-cells are anergic or rendered quiescent through the engagement of their programmed death receptor-1 (PD-1) with the PD1 ligand (PD-L1) Khandekar et al. Empowering the CD8^+^ T-cells with anti PD-1 or anti-PD-L1 immunotherapy would synergize with BCSC-directed therapy. Sridharan, Howard et al. have comprehensively reviewed various molecular targets in BCSCs that would render them susceptible for targeted therapy. In particular, targeting various molecular signaling receptors and downstream mediators that would reduce stemness and overcome chemoresistance of BCSCs were described. Sridharan, Robeson et al. have discovered a key vulnerability in BCSCs in that they are dependent on certain pro-survival factors such as survivin and Myeloid Cell Leukemia 1(MCL1) for their distinct survival advantages. Both survivin and MCL1 are also involved in chemoresistance (12). Also, oncotargets such as cyclin D1 and cyclin D3 are also key for BCSCs to maintain their clonogenicity. Interestingly, all these targets have one thing in common; the 5′-leader sequence of their mRNAs has a classical secondary structure that hinders facile mRNA translation by the ribosome. This problem can be rectified by the mRNA helicase eIF4A (13). By targeting this single enzyme eIF4A, the translation of a whole set of oncogenic mRNAs such as survivin, MCL1, cyclin D1, cyclin D3, mucin-1C, Rho kinase 1, STAT1 (this controls PD-L1 transcript level), and MDM2/HDM2 (controls wild type p53 level) will be obliterated (14). Importantly, the pluripotency transcription factors OCT4, SOX2 and NANOG were significantly downregulated when eIF4A was genetically ablated or pharmacologically targeted. This is the first report that targeting of eIF4A could downregulate BC stemness Sridharan, Robeson et al. Furthermore, the levels of ATP-binding cassette (ABC) transporters that export the xenobiotics were significantly reduced. This brings an important point in that targeting eIF4A not only controls BC stemness but also can overcome chemoresistance through downregulation of drug transporters Sridharan, Robeson et al. Importantly, eIF4A is distributed equally between BCSCs and non-BCSCs and so both cellular populations will be killed simultaneously leaving little chance for MRD, relapse and aggressive tumor. eIF4A is a promising new target in BCSCs and targeting eIF4A may help improve the clinical outcome. There is an ongoing clinical trial targeting eIF4A by eFT226 by Effector Therapeutics (NCT04092673) and hopefully beneficial for advanced cases of breast cancer.

As BCSCs play a role in drug tolerance and resistance, targeting the plasticity may lead to a profound and more durable response. A combinatorial treatment approach may overcome the multidrug resistance (MDR) encountered in the clinic. During cytotoxic and targeted therapies, activation of phosphatidylinositol-3-kinase (PI3K), yes-associated protein (YAP) signaling, up regulation of anti-apoptotic MCL1, hepatocyte growth factor [HGF; secreted by the carcinoma associated fibroblasts (CAFs)], insulin-like growth factor receptor (IGFR), epidermal growth factor (EGF) receptor and AXL ligand are frequently observed leading to chemoresistance (15). Tumors (primary or metastatic) enriched in CAFs (tumor microenvironment—TME) is likely to secrete HGF (ligand for c-Met receptor), CXCL12 (ligand for chemokine receptor CXCR4), and these factors may contribute to chemoresistance. Importantly, the difference in the TME between primary and the metastatic lesions should be considered as it may also dictate and produce diverse clinical outcomes. Based on biopsy results of such lesions, the level of PD-L1, PD-1 may also be assessed and appropriate co-targeted therapy directed toward cellular TME, BCSCs, and non-BCSCs should be devised. Combined targeting of these pathways along with a known targeted therapy may produce a better objective treatment response in containing BCSC and non-BCSC cell populations.

## Author Contributions

DR composed, edited, and finalized the editorial. AT, VT, and JR reviewed and finalized the editorial.

### Conflict of Interest

The authors declare that the research was conducted in the absence of any commercial or financial relationships that could be construed as a potential conflict of interest.
